# Particle packing into loose networks for tough and sticky composite gels

**DOI:** 10.1038/s41598-020-74355-8

**Published:** 2020-10-14

**Authors:** Taka-Aki Asoh, Tatsuya Yamamoto, Hiroshi Uyama

**Affiliations:** Department of Applied Chemistry, Graduate School of Engineering, Osaka University, 2-1 Yamadaoka, Suita, Osaka 565-0871 Japan

**Keywords:** Chemistry, Materials science

## Abstract

Hydrogel is an attractive material, but its application is limited due to its low mechanical strength. In this study, a tough composite gel could be prepared by synthesizing polymer particles within a polymer network having relatively loose cross-linking. Since the polymer network acts as a dispersion stabilizer during the synthesis of the hydrophobic polymer particles, a large amount of particles could be introduced into the gel without agglomeration. It was suggested that the high level of toughness was induced by the adsorption and desorption of the polymer chains on the surface of the finely packed particles. By using a stimuli-responsive polymer network, elasticity and plasticity of composite gels could be controlled in response to external stimuli, and adhesion on the gel surface could also be modulated.

## Introduction

Hydrogel, a water-containing material with cross-linked hydrophilic polymers, is attracting attention as a next-generation functional material^1–5^. However, general hydrogels have low mechanical strength due to their high swelling property and non-uniform structure, which is an obstacle to their wider application and development. In order to develop a gel with high strength and toughness that overcomes this drawback, research and development has been conducted for devising effective hydrogel molecular designs^6–11^. Typical examples include the development of a slide-ring gel^8^, a tetra-poly (ethylene glycol) (PEG) gel^9^, a double network (DN) gel^10^, and a nanocomposite gel^11^. A movable cross-linking point is introduced in the slide-ring gel, while the tetra-PEG gel has a uniform network structure stress evenly dispersed throughout the gel, and there is a suppression of cracks. DN gels form an interpenetrating network structure from a hard and brittle polymer network and a soft and extending polymer network, and nanocomposite gels combine clay as multifunctional cross-linking points to suppress the progress of cracks by stress yielding. Incorporating a molecular design that suppresses the generation and progress of internal cracks in response to applied stress is extremely important in achieving high toughness in hydrogels^12,13^.

From the viewpoint of increasing the strength and toughness of materials, much research has been conducted on composite materials in which a filler serving as a reinforcing agent is dispersed in a matrix^14^. In particular, fiber reinforced plastics^15^ are being developed and put to practical use in homes/construction, sports equipment, automobiles, aviation/space, etc. as substitutes for metals, because of characteristics such as their light weight and high strength. The use of organic or inorganic particles, fibers, graphene oxide, etc. as fillers in composite gels using hydrogel as a matrix has also been investigated, and high toughness has been achieved regardless of the type, shape, and size of fillers^16–21^.

Generally, a composite gel can be prepared by forming a polymer network of gel in the presence of a filler. The higher the amount of filler in the matrix, the higher the expected toughness of the composite gel. However, as the concentration of the filler in the gelling solution increases, the surface area of the interface between the filler and the solution increases. The interface is reduced by aggregation of the fillers, so that the stabilizing effect of the filler is diminished and the dispersibility decreases. It has been reported as a result, that the internal structure of the composite gel tends to be non-uniform and brittle^22^. Therefore, a major issue in producing a high toughness composite gel is how to both highly disperse and highly fill the filler.

**Figure 1:**
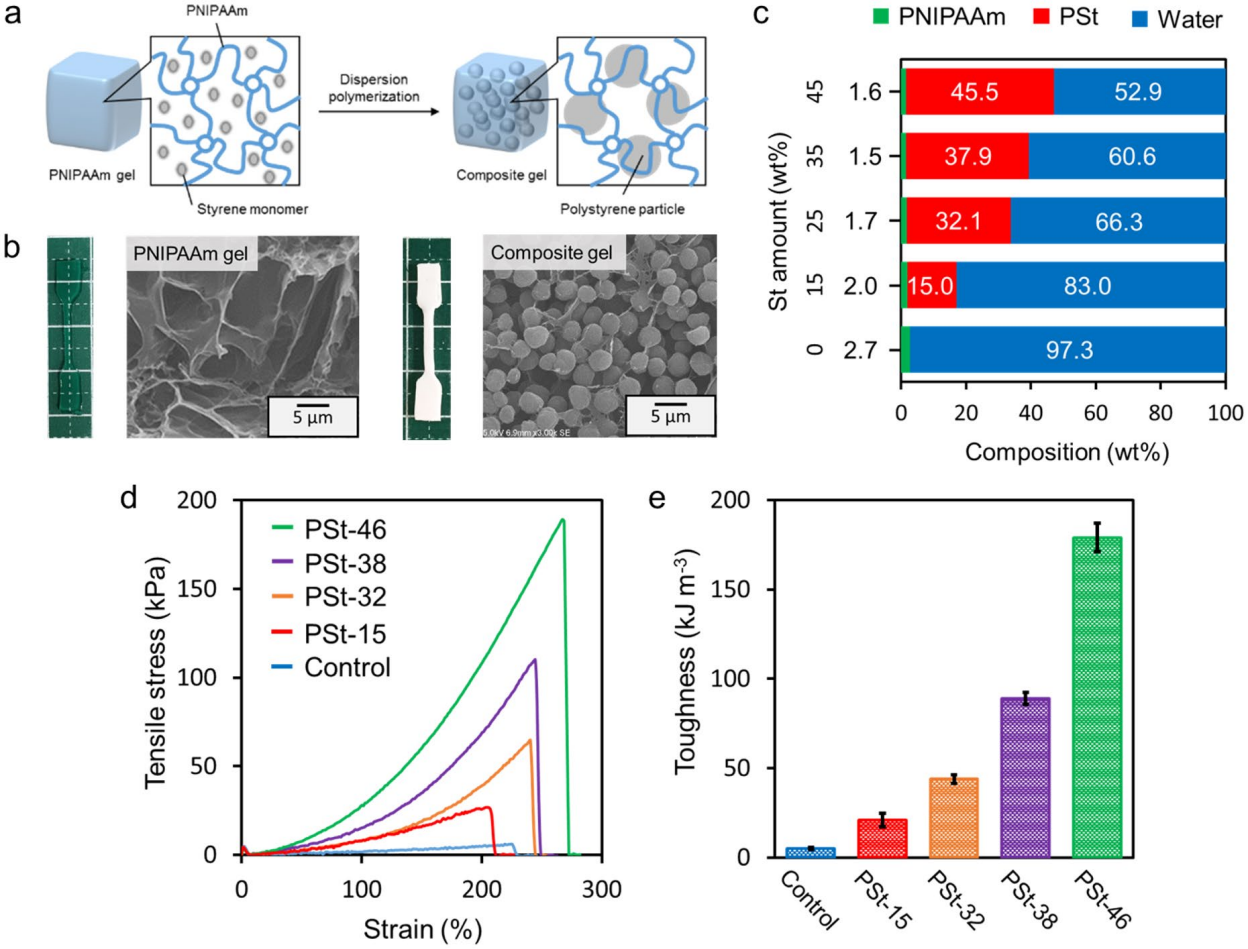
(**a**) Preparation of composite gels by synthesizing polymer particles within a polymer network having relatively loose cross-linking. (**b**) Macroscopic and microscopic images of PNIPAAm and composite gels. (**c**) Chemical composition of composite gels prepared by changing the St concentration in feed. (**d**) Typical stress–strain curves during tensile test (tensile speed: 6 mm s^−1^) and (**e**) toughness of composite gels.

In this study, we prepared a tough gel by filling a large amount of polymer particles into a hydrogel with an extremely low degree of cross-linking (Fig. 1a). Hydrogels with lower cross-linking ratios can reduce the stress-concentration to non-uniform domains, to the utmost limit; but such gels have a high water content and a low elastic modulus, and are difficult to be applied to applications requiring mechanical strength. We focused on dispersion polymerization, which is typically used for synthesis of polymer particles. Dispersion polymerization is polymerization in which a hydrophobic monomer is dissolved in a selective solvent in the presence of a small amount of a polymer dispersant, resulting in the polymer precipitating and forming particles during the polymerization^23,24^. The polymer dispersant is likely to be dissolved in the polymerization solvent and adsorbed at the particle interface. It was also possible that the water-soluble polymer network of the hydrogel with a low cross-linking ratio could, like the water-soluble polymer chain, also play the role of a dispersant in the dispersion polymerization. It was also assumed that when a polymer network was used as a dispersant, a large amount of hydrophobic particles could be packed without agglomeration due to the high steric stabilizing effect from the elastic network. In this study, we aimed to make a tough hydrogel by forming a large amount of hydrophobic particles without agglomeration, and by dispersing and polymerizing the hydrophobic monomers inside the hydrogel.

## Results and discussion

A Poly(*N*-isopropylacrylamide)-poly(styrene) (PNIPAAm-PSt) composite gel was prepared by dispersion polymerization of styrene (St) inside the PNIPAAm gel with lower cross-linking density (Cross-linker content are 0.2 mol% to NIPAAm) (Fig. 1a). As shown in Fig. 1b, following polymerization of St, the transparent PNIPAAm gel became cloudy, indicating that polystyrene (PSt) particles were formed inside the PNIPAAm gel. Scanning electron microscopy (SEM), revealed only the PNIPAAm skeleton in the PNIPAAm gel, whereas in the composite gel, a structure was observed in the PNIPAAm gel with PSt particles packed inside it. No aggregation of PSt particles was observed. This indicates that the PNIPAAm chain acted as a dispersant during the dispersion polymerization of St. The chemical percent composition (by mass) of the composite gels was calculated from the weight of PNIPAAm and the composite gel before and after lyophilization (Fig. 1c). It was found that the water content decreased, and the PSt content increased to 46 wt% at the maximum, as the amount of St in the feed ratio increased. On the other hand, the water content with respect to PNIPAAm was almost unchanged before and after the incorporation of PSt particles. Therefore, the gel volume increased with increasing PSt content, up to a maximum of 1.76 times.

The mechanical strength of PNIPAAm and composite gels was evaluated by a tensile test (Fig. 1d). The PNIPAAm gel had a breaking strength of 5 kPa; whereas the fracture strength of the composite gels increased as the amount of PSt increased, reaching a maximum of 188 kPa for PSt-46. The improvement of the fracture strength by the PSt filler is the same as the effect of a general filler. Interestingly, the maximum strain value did not decrease after the PSt incorporation. This result suggests that PSt particles are present in the PNIPAAm gel without agglomeration, despite their high loading of 46%. Due to improved fracture strength without compromised elongation, the toughness of composite gels increased by up to 36 times that of PNIPAAm gels (Fig. 1e). The extremely low strength PNIPAAm gel ruptured upon exertion of a very weak force, whereas the composite gel showed a significant increase in fracture strength, suggesting that stress was dispersed by energy dissipation. Therefore, it was suggested that by synthesizing PSt particles inside the PNIPAAm gel, a structure that dissipates energy is formed inside the gel. As shown in Fig. S1, this property was exhibited even when the crosslinking degree of the gel was increased from 0.2 to 3 mol%, although increasing the degree of cross-linking reduced the dispersibility of the polymer network and resulted in non-uniform particle size. Furthermore, even when different kinds of hydrogels and hydrophobic particles are used, the hydrophobic particles can be filled without agglomeration and the hydrogel demonstrates high toughness (Figs. S2 and S3)—indicating that the interaction between the polymer network and the particles contributes to the toughening of the composite gels. In recent years, self-growing gels in which broken parts are polymerized have been reported^25^, and it is also interesting that mechanical strength can be improved posteriorly.

**Figure 2:**
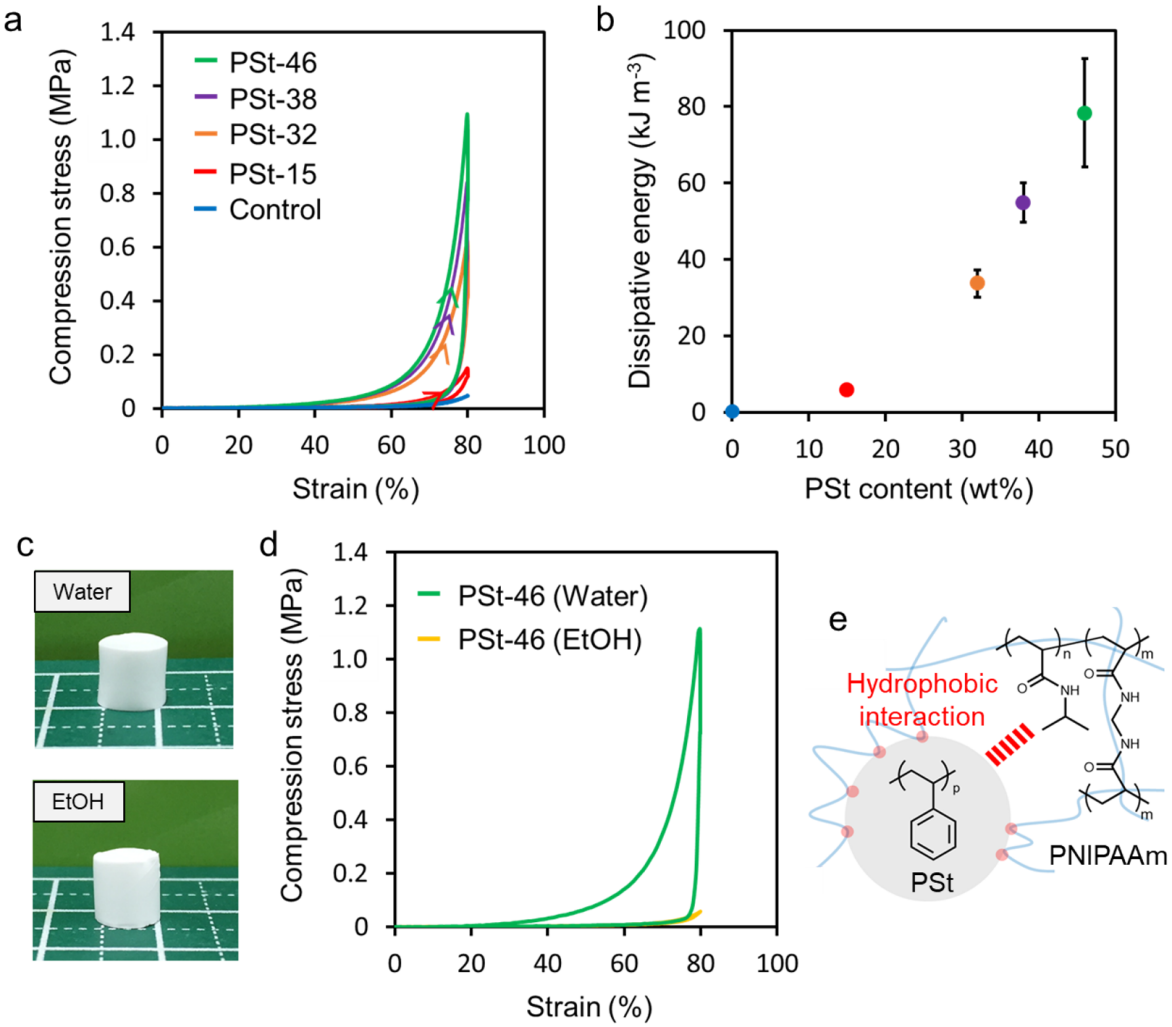
(**a**) Loading–unloading experiment of composite gels by compression and (**b**) dissipative energy calculated by loading–unloading curves (test speed: 5 mm min^−1^). (**c**) Macroscopic images and (**d**) loading–unloading curves of composite gels swelled in water and EtOH. (**e**) Proposed mechanism for generation of toughness by adsorption of polymer chains on the particle surfaces.

Energy dissipation of the composite gels was evaluated by the loading–unloading test (Fig. 2a). In the stress–strain curve, the PNIPAAm gel did not show the hysteresis. However, hysteresis loops were confirmed for the composite gels, indicating that their structures dissipate energy. In the loading–unloading test, unloading was performed at the same speed immediately after 80% compression without waiting time. Since the PNIPAAm gel and the composite gel recovered to their original shape after unloading, it is considered that the energy dissipation is not due to the destruction of the skeletal structure of the PNIPAAm gel. As shown in Fig. 2b, the energy dissipation increased with increasing PSt content. These results also suggested that the interaction between PSt particles and PNIPAAm chains contributes to the toughness of the composite gel by dissipating its energy. Water and ethanol were poor and good solvents respectively for PSt and PNIPAAm, respectively (Fig. 2c). The compressive strength and energy dissipation of the composite gel swollen with water and ethanol were subsequently evaluated (Fig. 2d). The composite gel (PSt-46) swollen in water was very tough, while its compressive strength and energy dissipation when swollen in ethanol showed very low values of 0.06 MPa and 1.5 kJ m^−3^, respectively. The interaction between the particles and PNIPAAm for toughness is considered to be hydrophobic because of the difference in results from the experiments in water and ethanol (Fig. 2e). The hydrophobic interaction between PSt particles and the PNIPAAm chain was reduced in ethanol, but in water, adsorption of PNIPAAm chains on the surface of PSt particles contributed to energy dissipation.

**Figure 3:**
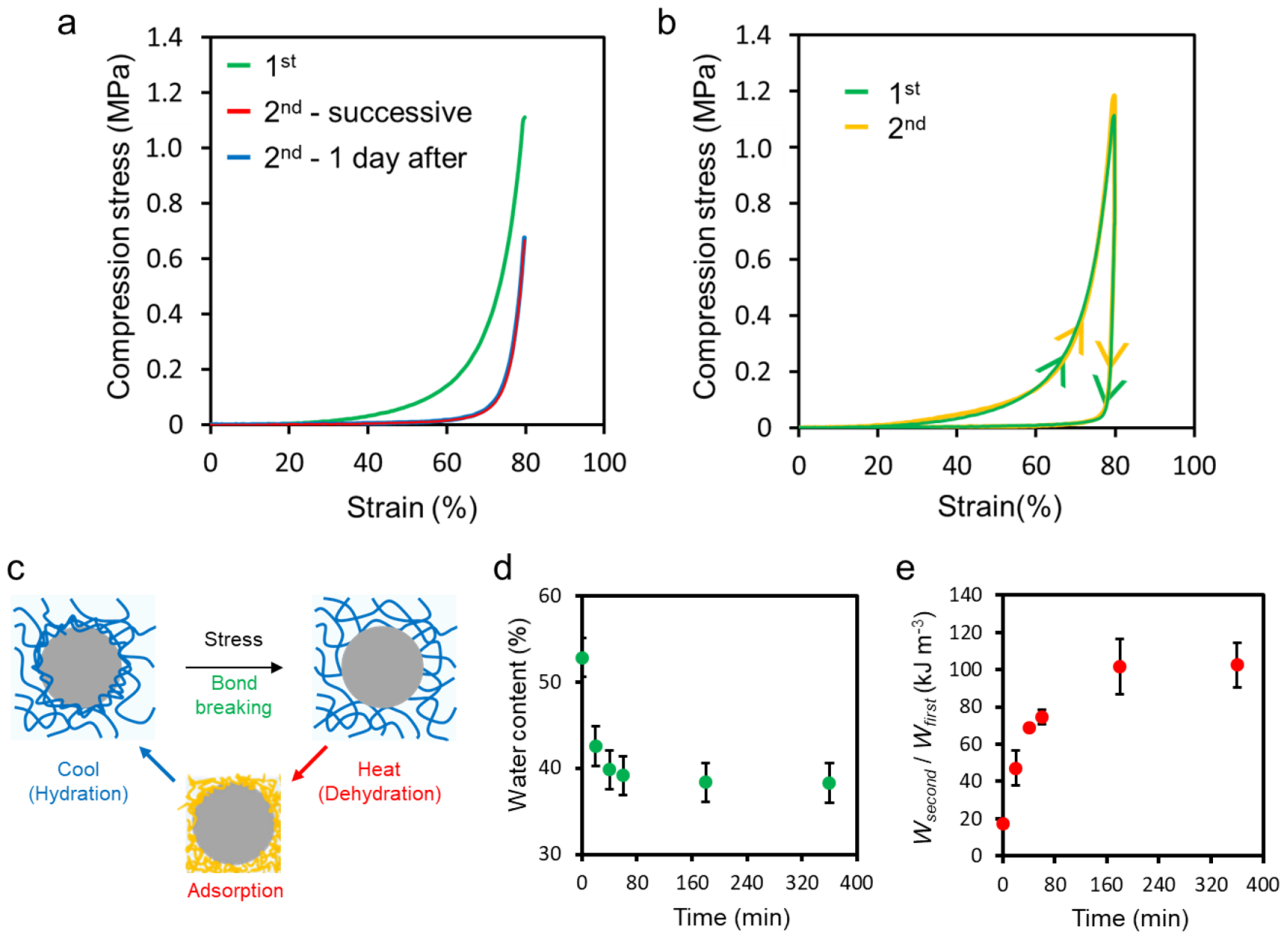
Stress–strain curves of composite gels for 1st and 2nd compression (**a**) before and (**b**) after heating–cooling process. (**c**) Reversible adsorption of polymer chain on the particle surfaces by applying a stress and shrinking process. (**d**) Water content and (**e**) recovery of dissipative energy as a function of incubation time at 50 °C.

Although the compressive strength was 1.1 MPa at 80% compression in the first compression, it decreased to 0.7 MPa when the second compression test was continuously performed (Fig. 3a). Moreover, the compressive strength was 0.7 MPa when subjected to the same test after being left standing for 1 day in water after the first compression. These results show that the structure that was destroyed during compression could not be recovered only by allowing it to stand in water. However, the stress–strain curves were similar to the first curve when a second loading–unloading was performed by heating and cooling the gel after the first compression (Fig. 3b). This means that the structure destroyed during compression was reconstructed by heating and cooling processes. PNIPAAm hydrogel is known as a thermoresponsive hydrogel. The PNIPAAm gels swelled up in water at temperatures below 32 °C, the so-called lower critical solution temperature (LCST); however, they shrunk with water release at temperatures above the LCST (Fig. S4A)^26,27^. Since a large amount of particles were filled in polymer networks, the shrinking of composite gels was inhibited by particles^19^, and the swelling ratio, *D*/*D*_0_, when the temperature was changed from 20 to 50 °C was suppressed to below 10%. However, similar to the PNIPAAm gel, the composite gel (PSt-46) showed discontinuous size change around 30–35 °C (Fig. S4B). It is reasoned that the PNIPAAm chain was dehydrated and became hydrophobic in water when the temperature increased, and hydrophobic PNIPAAm chains were re-adsorbed on the PSt surface to recover the toughness (Fig. 3c).

The recovery of energy dissipation after the first compression was evaluated as a function of the water content of composite gels. The water content decreased as the heating time of the composite gel at 50 °C increased, before reaching a constant value after 180 min (Fig. 3d). On the other hand, dissipative energy was increasingly recovered with heating time, and reached a constant value after 180 min (Fig. 3e). Since there was a correlation between the water content during heating and the recovery of energy dissipated after cooling, it was suggested that the amount of PNIPAAm chains adsorbed on the PSt particle surface increased with an increasing shrinkage of composite gels. The coil-globule transition of PNIPAAm is caused not only by temperature change but also by the composition of the solvent. Therefore, the recovery ratio of the dissipative energy when the composition of water and ethanol was changed were evaluated, it was shown that the dissipative energy was largely recovered at solvent composition where the gels was most shrunk (Fig. S5). Also, about the effect of the solvent, similar toughness switching is possible not only with temperature changes but also with the coil-globule transition in solvent changes. Adsorption of PNIPAAm chains on the surface of PSt was also supported by X-ray photoelectron spectroscopy (XPS) measurements (Fig. S6).

**Figure 4:**
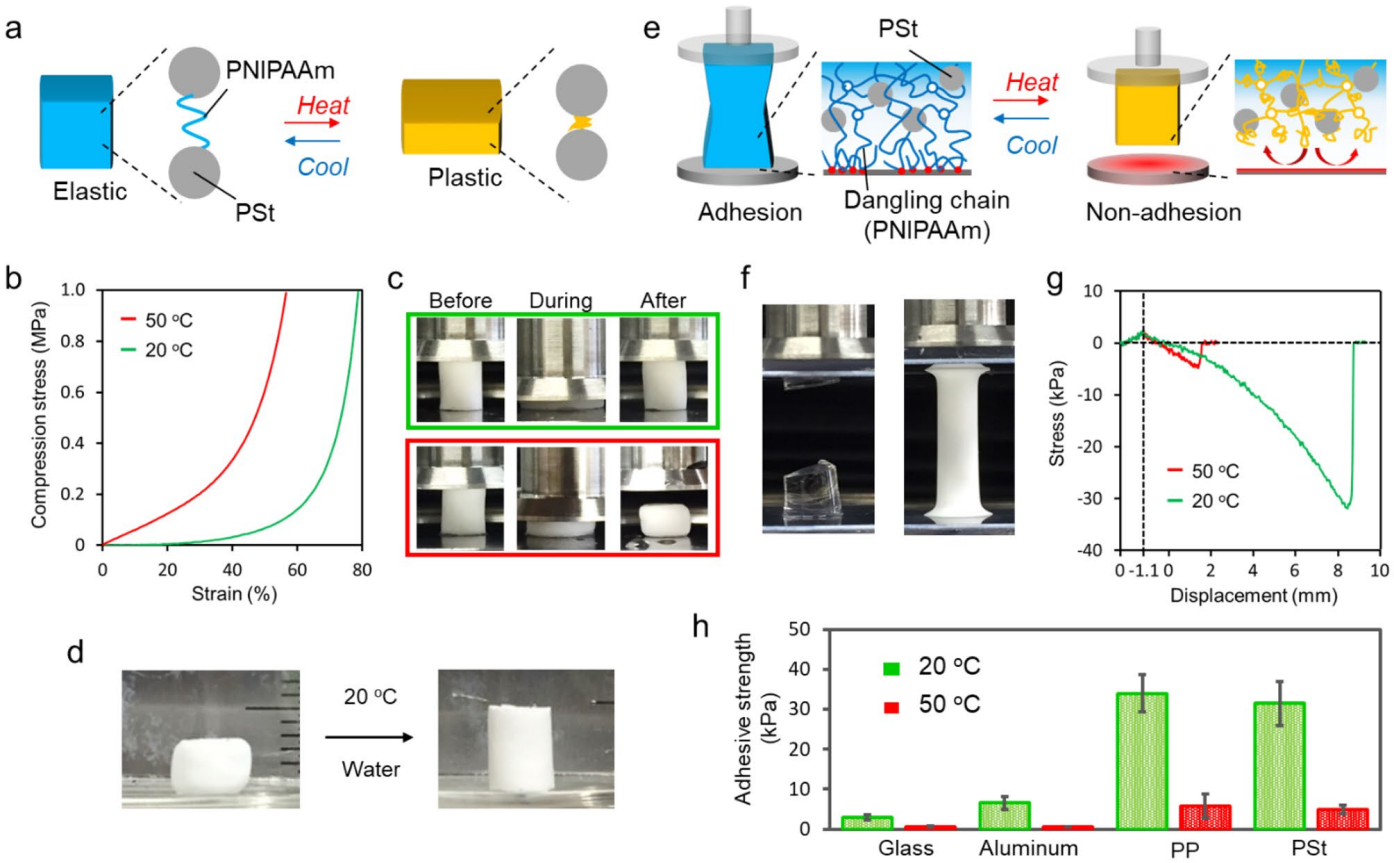
(**a**) Elastic–plastic modulation by temperature. (**b**) Stress–strain curves of composite gels at 20 and 50 °C and (**c**) images of composite gel during compression experiment. (**d**) By immersion into water at 20 °C, plastic deformed composite gels at 50 °C returned to original shape by swelling into water. (**e**) Adhesion control utilizing dangling chains on the surface of composite gels. (**f**) Image of PNIPAAm (left) and composite (right) gels during adhesion strength measurement. The PNIPAAm gel itself broke due to its low mechanical strength. (**g**) Adhesive strength of composite gels at 20 and 50 °C. (**h**) Adhesive strength of composite gels for glass, aluminum, PP, and PSt at 20 and 50 °C. Values is mean ± SD when three measurements are performed using different samples.

The change in the stiffness of composite gels (PSt-46) was evaluated by the compression test at 20 and 50 °C (Fig. 4a). As shown in Fig. 4b, the strain value when a stress of 1 MPa was applied at 20 °C was 78%, whereas it was 57% at 50 °C, indicating that the stiffness of the composite gel changed with the temperature change. The appearance of the composite gel during and after the compression test was then observed (Fig. 4c). It returned to its original shape at 20 °C after unloading, and showed elastic properties; while at 50 °C it did not return to its original shape and was plastically deformed. This was because at 50 °C, which is above the LCST of PNIPAAm, PNIPAAm formed a globular state and its elasticity disappeared. As a result, plasticity was predominantly observed. Interestingly, when the plastically deformed composite gel was immersed in 20 °C water, which is below LCST, the original shape was restored (Fig. 4d). From the above results, it was found that it is possible to control the elasticity and plasticity of the composite gel by changing the temperature.

Because of a lower cross-linking ratio, current tough composite gels have many dangling chains on the surface and show sticky properties^28–30^. In a general hydrogel, in order to exhibit such properties, it is necessary to create an extremely low degree of crosslinking. The low elastic modulus, therefore makes it is difficult to apply it to structural materials. By packing hydrophobic particles inside the gel having a low degree of cross-linking, it is possible to increase the toughness without impairing the surface properties due to the dangling chains, and it is possible to change the adhesion by utilizing the temperature responsiveness (Fig. 4e).

PNIPAAm gel demonstrated cohesive failure at a maximum adhesive strength of 3.2 kPa, because the gel itself broke due to its low mechanical strength (Fig. 4f; left). On the other hand, in composite gels (PSt-46), interfacial peeling occurred at the maximum adhesive strength of 31.5 kPa (Fig. 4f; right). This result indicates that composite gels have both the mobility of the polymer chains on the surface and the toughness of the bulk. Moreover, with increasing temperature, the adhesion strength was significantly reduced because dangling chains transformed into globule states (Fig. 4g). Subsequently, the adhesive strengths of different substrates were evaluated (Fig. 4h). The maximum adhesive strength was 3.0, 6.6, 33.0, and 31.5 kPa for glass, aluminum, polypropylene (PP), and PSt, respectively. It has been found that a hydrophobic surface such as PP or PSt has a higher adhesive strength than a hydrophilic surface such as glass or aluminum. For all substrates, the adhesive strength can be controlled by temperature; the maximum adhesive strength at 50 °C decreased to less than one-sixth of 20 °C. It was possible to control the adhesion of the composite gels by the temperature.

### Conclusion

A tough and surface-adhesive composite gel was prepared by synthesizing a large amount of particles within a loosely cross-linked polymer network. The polymer network played the role of a dispersant during the precipitation polymerization, and was able to be synthesized without agglomeration of particles inside the hydrogel. It was proposed that the energy is dissipated by the detachment of polymer chains from the particle surface when stress is applied, and the toughness of the composite gel is improved. The toughness therefore was restored by re-adsorption of the polymer chains on the surface of the particles. It was possible to reversibly control the elasticity and plasticity of the composite gel by changing the temperature using a temperature-responsive polymer. Since the particles were filled subsequently, it was found that the adhesiveness of the surface derived from the low cross-linking network was retained and the adhesiveness was controllable by temperature change. The novel synthesis strategy in this study for improving the toughness of a composite gel offers a new design guideline for the synthesis of polymer gels with high mechanical strength and excellent surface properties.

## Methods

### Materials

*N*,*N*-Dimethylacrylamide (DMAAm), Styrene (St), and methyl methacrylate (MMA) was purchased from Wako Pure Chemical Industries and used after distillation. *N*-isopropylacrylamide and azobis(isobutyronitrile) was purchased from Wako Pure Chemical Industries and used after recrystallization. Ammonium persulfate (APS), *N*,*N*′-methylenebis(acrylamide) (MBAAm), *N*,*N*,*N*’,*N*’-tetramethylethlenediamine (TEMED) were purchased from Wako Pure Chemical Industries (Osaka, Japan) and used as purchased without further purification.

### Preparation of composite gels

Poly(*N*-isopropylacrylamide) (PNIPAAm) hydrogels were prepared by free-radical polymerization of NIPAAm (0. 7 mol L^−1^) in the presence of *N*,*N*′-methylenebis(acrylamide) (MBAAm) (0.2, 1.0, 3.0 mol% to NIPAAm) at 4 °C by using ammonium persulfate (APS) and *N,N,N',N*′-tetramethylethane-1,2-diamine (TEMED) as redox initiator. After polymerization, water inside PNIPAAm gels was replaced with ethanol (EtOH). The EtOH swollen PNIPAAm gel was immersed into an EtOH solution in which a predetermined amount of styrene (St) and azobis(isobutyronitrile) (AIBN) initiator were dissolved, and kept at 4 °C for 24 h. Precipitation polymerization of St was carried out inside PNIPAAm gels at 65 °C for 24 h after degassing. The composite gels obtained were washed by excess amounts of EtOH for removal of unreacted St and then replaced into water. The composite gels were coded as PSt-X (X wt%: PSt content) according to the PSt content of the chemical composition of the composite gels.

## Supplementary information

Supplementary Information.
