# Supplementary material for: Particle packing into loose networks for tough and sticky composite gels
Source: Sci Rep. 2020 Oct 14;10:17173. doi: 10.1038/s41598-020-74355-8 (PMC7560882; doi:10.1038/s41598-020-74355-8)
Supplement: Supplementary file 1 — Supplementary Information. [file 41598_2020_74355_MOESM1_ESM.docx]

**Supporting Information**

Particle Packing into Loose Networks for Tough and Sticky Composite Gels

Taka-Aki Asoh,* Tatsuya Yamamoto, Hiroshi Uyama

**Effect of cross-linking ratio of composite gels**

**
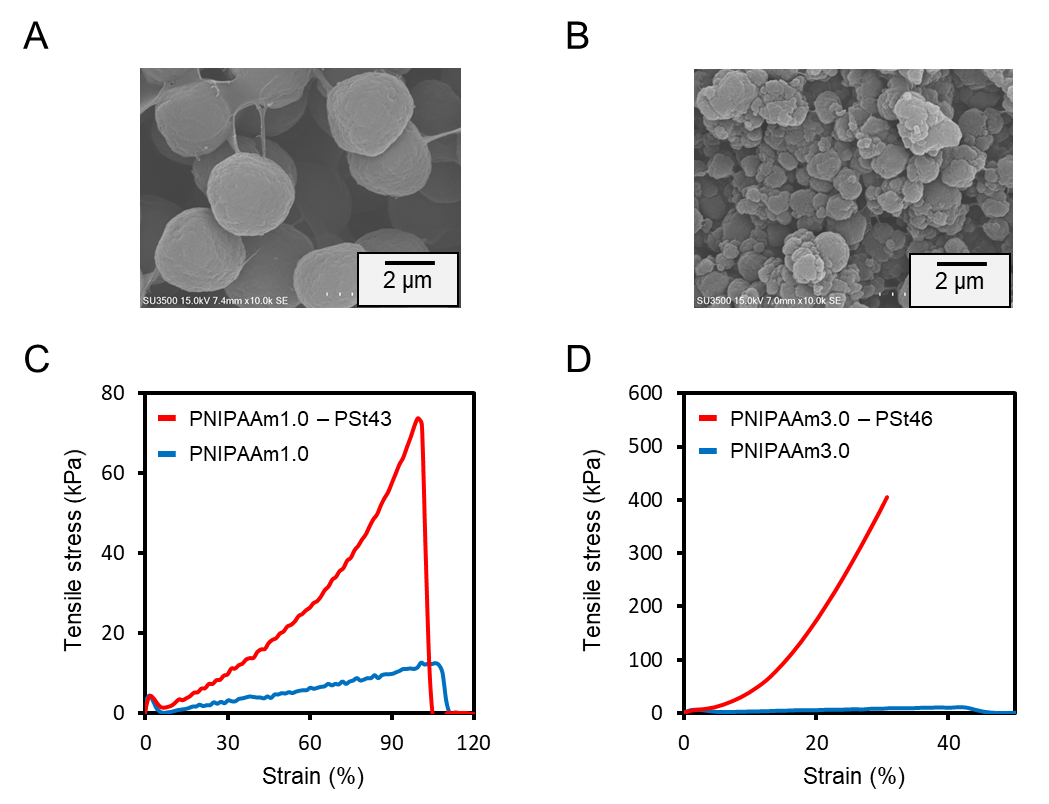
**

**Figure S1**. SEM images of (A) PNIPAAm1.0-PSt43 and (B) PNIPAAm3.0-PSt-46. Tensile stress-strain curves of (C) PNIPAAm1.0, PNIPAAm1.0-PSt-43 and (D) PNIPAAm3.0, PNIPAAm 3.0-PSt-43.

**Preparation of PNIPAAm-poly(methyl methacrylate) composite gels**

Dispersion polymerization of MMA was performed inside the PNIPAAm gels (cross-linking ratio is 0.2 mol%) to prepare a PNIPAAm-PMMA composite gels. The PNIPAAm gels before composite with PMMA particles had a breaking strength of 5 kPa, whereas the composite gels had an improved breaking strength of up to 287 kPa. The toughness was also greatly improved and increased up to 80 times. As in the case of PSt particles, PMMA particles can be formed without agglomeration by performing dispersion polymerization of MMA inside the PNIPAAm gels.


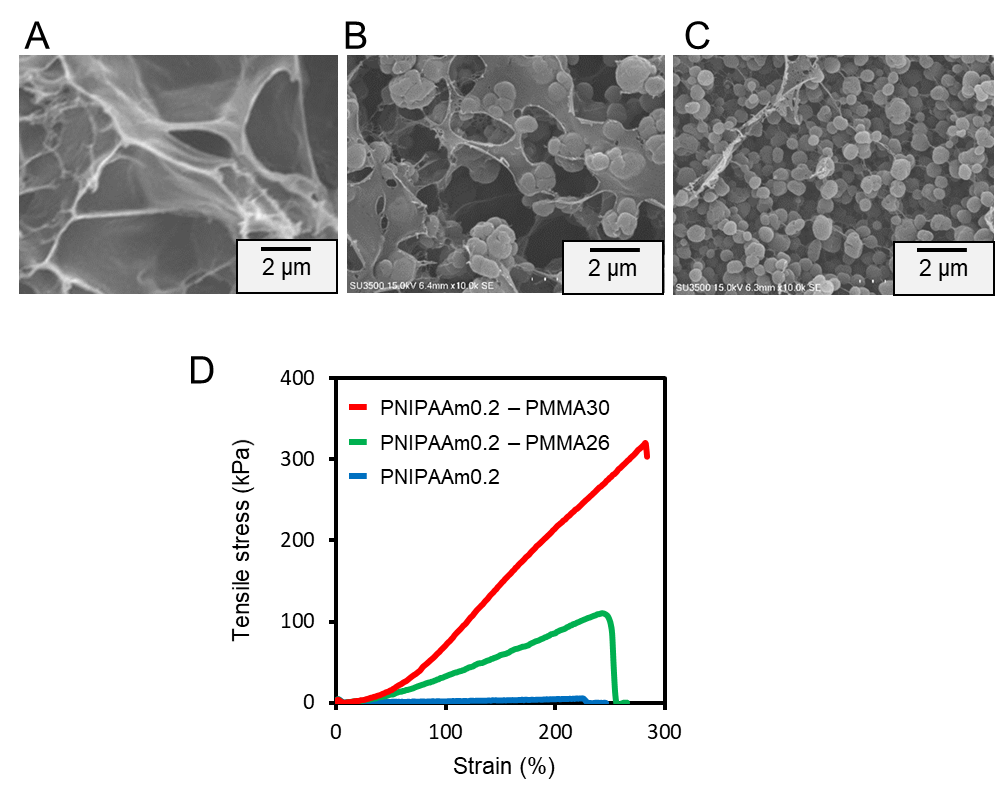


**Figure S2**. SEM images of (A) PNIPAAm0.2, (B) PNIPAAm0.2-PMMA-26 and (C) PNIPAAAm0.2-PMMA-30, and (D) tensile stress-strain curves.

**Preparation of poly(*N*,*N*-dimethylacrylamide)-PSt composite gels**

Dispersion polymerization of St was performed inside the poly(*N*,*N*-dimethylacrylamide) (PDMAAm) gels (cross-linking ratio is 0.05 mol%) to prepare a PDMAAm-PSt composite gels. The breaking strength of the PDMAAm gels before PSt particle composite was 15 kPa, whereas the breaking strength of the composite gels was improved to a maximum of 84 kPa. Because there was no change in elongation before and after PSt particle composite, the toughness was also greatly improved and increased up to 4 times.


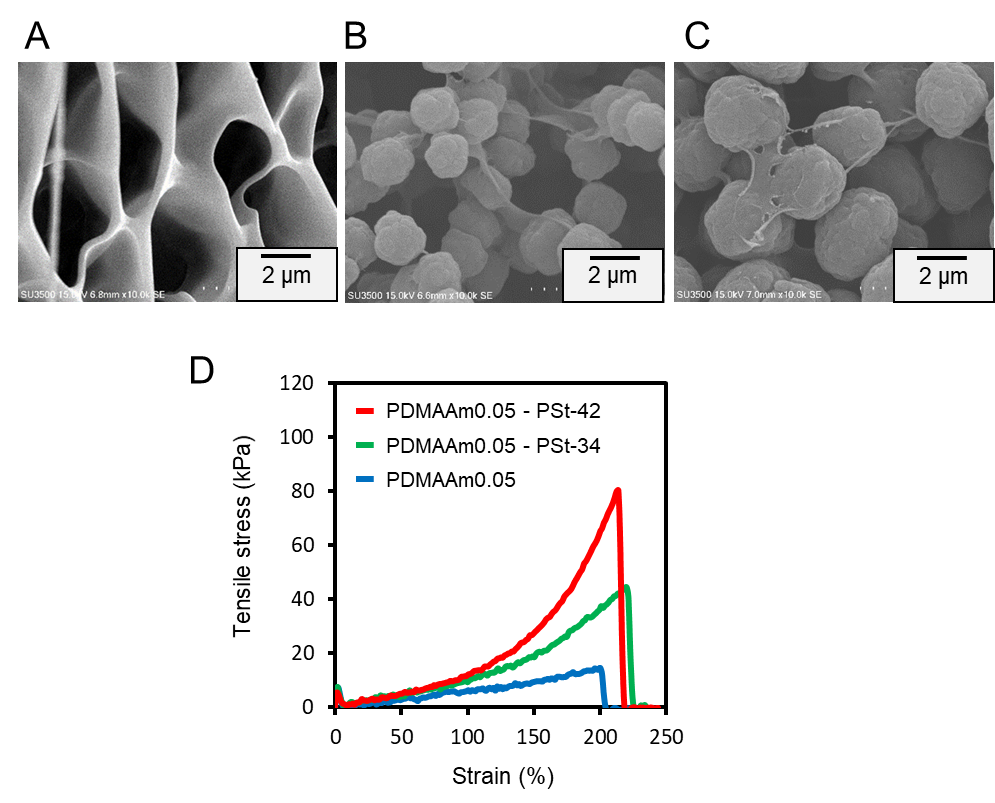


**Figure S3**. SEM images of (A) PDMAAm0.2, (B) PDMAAm0.2-PSt-34 and (C) PDMAAm0.2-PSt-42, and (D) tensile stress-strain curves.

**Temperature responsive properties**

**
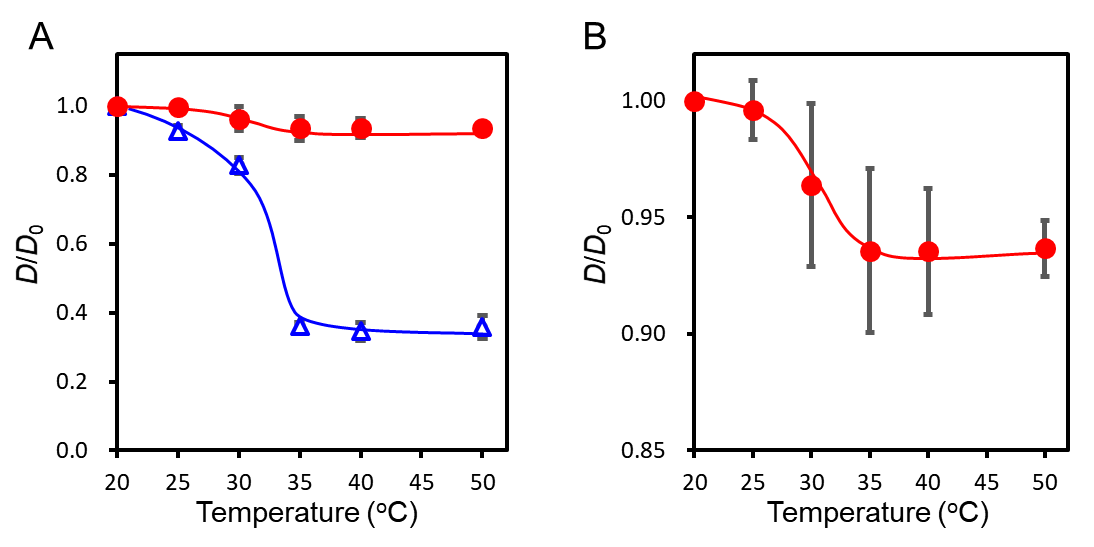
**

**Figure S4** (A) Swelling ratio, *D*/*D*_0_, of the PNIPAAm 0.2 (triangle) and PSt-46 (circle) gels as a function of temperature. The swelling ratios were calculated from the ratio between the diameter (*D*) at the corresponding temperature and the diameter of at 20^o^C (*D*_0_). (B) Enlarged figure of PSt-46.

**Recovery of dissipative energy by co-nonsolvent**


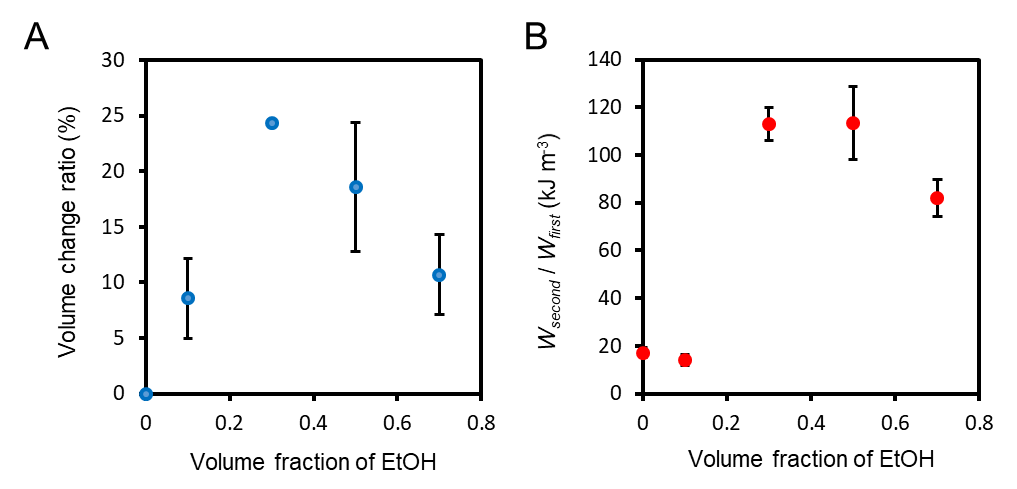


**Figure S5**. (A) Volume change ratio and (B) recovery of dissipative energy of composite gels as a function of volume fraction of EtOH in water.

**XPS measurement**

The interaction between PNIPAAm and PSt was evaluated by XPS measurement. PSt substrate was immersed into PNIPAAm ethanoic solution and aqueous solution at 20 and 50 ^o^C, and measured the PSt substrate after washing. No nitrogen (N) atom was detected in PSt, whereas a peak of N atom was detected in PSt-PNIPAAm at 20 ^o^C in water. This suggests that PNIPAAm is adsorbed on the PSt surface due to the hydrophobic interaction between PNIPAAm and PSt in the aqueous environment. The number of N atoms at 20 and 50 ^o^C in water was 4.6 x 10^4^ and 6.3 x 10^4^, respectively. It is considered that the adsorption of PNIPAAm to the PSt substrate was induced during the phase transition of PNIPAAm. The number of N atoms of PSt-PNIPAAm at 20 ^o^C in EtOH was 0.5 x 10^4^, which was lower than those of in water. It is considered that the hydrophobic interaction between the PNIPAAm and PSt became smaller in the EtOH environment than in the water environment.


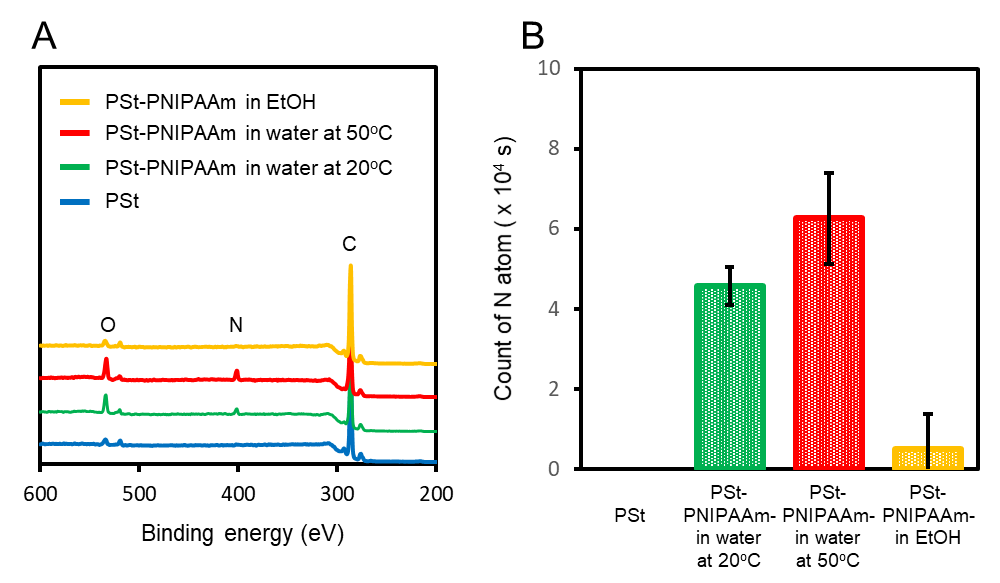


**Figure S6**. XPS wide scan spectra of PSt, PSt-PNIPAAm in water at 20 and 50^o^C, PSt-PNIPAAm in EtOH.
